# Does a home-based interview with a chronically ill patient help medical students become more patient-centred? A randomised controlled trial

**DOI:** 10.1186/s12909-020-02136-y

**Published:** 2020-07-11

**Authors:** Michael Harris, Anna-Lea Camenzind, Rita Fankhauser, Sven Streit, Roman Hari

**Affiliations:** 1 Institute of Primary Health Care (BIHAM), University of Bern, Bern, Switzerland; 2 College of Medicine & Health, University of Exeter, Exeter, UK; 3 Gore Cottage, Emborough, Radstock, BA3 4SJ UK

**Keywords:** Medical professionalism, Patient-centred care, Primary health care, Undergraduate medical education

## Abstract

**Background:**

While patient-centred care improves patient outcomes, studies have shown that medical students become less patient-centred with time, so it is crucial to devise interventions that prevent this. We sought to determine whether first-year medical students who had a structured home-based interview with a chronically ill patient became more patient-centred than those who had a sham intervention.

**Methods:**

This randomised controlled trial assigned first-year students from the University of Bern, Switzerland, to either an interview with a chronically ill patient at the patient’s home or to a sham comparator. We used the PPOS-D12 questionnaire to measure students’ levels of patient-centredness at baseline, and changes in these levels during their longitudinal primary care clerkship.

**Results:**

A total of 317 students participated. Patient-centred attitudes increased during the study. A home-based interview with a chronically ill patient had no additional effect. Being female and having been exposed to patients before medical school were associated with being more patient-centred at baseline. Students were less patient-centred than their General Practitioner teachers.

**Conclusions:**

A structured, home-based interview with a chronically ill patient did not change students’ patient-centred attitudes, so cannot be recommended as a way to influence those attitudes. However, patient-centred attitudes increased during the students’ first year of study, possibly because of their longitudinal primary care clerkship.

**Trial registration:**

Clinicaltrials.gov reference: NCT03722810, registered 29th October 2018.

## Background

In patient-centred practice, clinicians and patients share control of the consultation and of decisions about management of health problems, and physicians see their patients in a social context [1]. Patient-centred clinicians take the patient’s preferences and desires into account and integrate them into a care plan that is negotiated between them; they seek to understand patients as well as their diseases, to ‘see the illness through the patient’s eyes’ [2]. Patient-centred care is associated with favourable biomedical, psychological and social outcomes [3], can increase patient satisfaction [1], and can encourage patients to share useful information [4]. The degree to which health care is patient-centred can be viewed as a measure of its quality [5].

While medical students feel that a patient-centred approach is an important part of medical professionalism [6], longitudinal studies in the USA [7], Greece [8] and South Africa [9] found that students grew less patient-centred during their undergraduate course. A Japanese study found that resident physicians at a university hospital also became less patient-centred over the course of the year [10]. However, increasing patient-centred practice through interventions is challenging. One study that assessed the effect of interpersonal skills lectures and teaching on practitioner-patient interaction found that this did not significantly increase patient-centred practice in first-year undergraduates [11]. Early patient contact may, however, be effective: a study investigating the perspectives of tutors and students on the increasing students’ awareness of professionalism in the early years at a Scottish university found that early patient contact experiences were particularly important [12]. The study also found that learning activities that promoted critical reflection had a positive effect, and that role models contributed powerfully to students’ learning and identity formation.

Many medical schools have redesigned their curricula to help their students deliver person-centred healthcare [13]. Some have used home visits for this: home visits have been found to teach students how individualised care helps to meet chronically ill patients’ needs, and students have reported that the experience heightens their empathy and sensitivity towards these patients [14]. Student home visits result in more positive attitudes to their patients, in that they increase behaviours and attitudes that promote patients’ and families’ best interests [15], and they have been found to be a time-effective way of fostering students’ professional growth [16]. The timing of students’ home visits during their undergraduate career is also important, with evidence that they have a stronger positive effect on students’ attitudes when performed earlier [17]. Home visits to chronically ill patients can have a profound effect: there is evidence that they continue to positively influence students over subsequent years, with some reporting vivid memories of patients they had seen, and that when caring for patients 2 years later they were still applying the lessons they had learned [14].

To encourage students to take a patient-centred approach, we therefore designed a teaching module that took these factors into account. First-year medical students were assigned to visit a chronically ill patient in their own home and conduct a structured in-depth interview that used open-ended questions to elicit the patient’s narrative, and then participate in a structured debriefing with their General Practitioner (GP) teacher. To assess the effectiveness of this module, we conducted a randomised controlled trial to investigate whether these students subsequently had more patient-centred attitudes than those who had a sham intervention.

## Methods

### Design

This randomised controlled trial was conducted at the University of Bern, Switzerland and affiliated GP teaching practices between September 2018 (recruitment) and June 2019 (end of follow-up). All first-year medical students were randomly assigned to either the intervention arm or a sham comparator, which took place during one of the last days of their longitudinal clerkship in primary care.

### Interventions

The active intervention was a structured, in-depth interview with a chronically ill patient that had been chosen by the student’s allocated GP teacher. Each GP teacher was asked to select a patient who had one of the four chronic diseases with the highest disability-adjusted life years (DALY) scores in Switzerland: ischaemic heart disease, low back pain, major depressive disorder and chronic obstructive pulmonary disease [18]. GP teachers and students were told that these interviews needed to be unaccompanied and conducted at patients’ own homes. A structured debriefing discussion with the GP teacher followed each interview.

The comparator was a sham intervention in which GP teachers were asked to give students time to read a document that taught students about consultation skills and asked questions that the students needed to discuss with their GP teachers. This self-study document was designed to provide educational value and complement the University’s consultation skills teaching. We selected this as a sham intervention because there was existing evidence that teaching students interpersonal skills and training them in practitioner-patient interaction did not make them more patient-centred [11].

In their first months of medical school, students received seven half-days of training in primary care clerkships. To this, we added the half-day assigned for the study interventions. Students had no other patient-related experience or training during the observation period, which primarily consisted of teaching in the basic medical sciences.

### Outcome measures

We assessed patient-centred attitudes with the Patient-Practitioner Orientation Scale (PPOS) [19]. The original English-language version was designed to differentiate between patient-centred and doctor-centred attitudes [8], and is used to assess attitude changes in medical student cohorts as they progress through the clinical curriculum. We used a German-language version, PPOS-D12, which has been validated as an instrument for assessing patient-centred attitudes among medical students in German-speaking countries [20] (English translation given in Additional file 1).

The primary outcome measure was how the change in students’ PPOS-D12 scores over the time of their six-month primary care attachment compared between the active and sham intervention groups. Secondary outcomes were overall change in students’ PPOS-D12 scores during course of the study, and the effect on their baseline PPOS-D12 scores of: students’ gender; whether they had previously studied another subject as an undergraduate; pre-medical school contact with patients; and prior experience of chronic illness in the participants themselves or their close relatives and friends.

Experiences of their mentors’ behaviours, both positive and negative, shape medical students’ perceptions of the profession’s values [21–23]. We therefore hypothesised that students’ patient-centred attitudes might change to become more similar with those of their GP teachers, and conducted a nested study to compare changes in the PPOS-D12 scores of students with those of their GP teachers.

### Data collection

At the start of their first academic year, before they began their six-month primary care attachment, students completed an online survey that asked for demographic information and then administered the PPOS-D12.

After the interventions, students completed a second online questionnaire that asked which intervention they had been allocated to and which they had actually received, and again administered the PPOS-D12. Students who did not complete the survey were sent multiple reminders. Their GP teachers also completed an online PPOS-D12 survey after their attachment to the student had ended. Surveys were not anonymous, so that we could link student and teacher data.

**Fig. 1:**
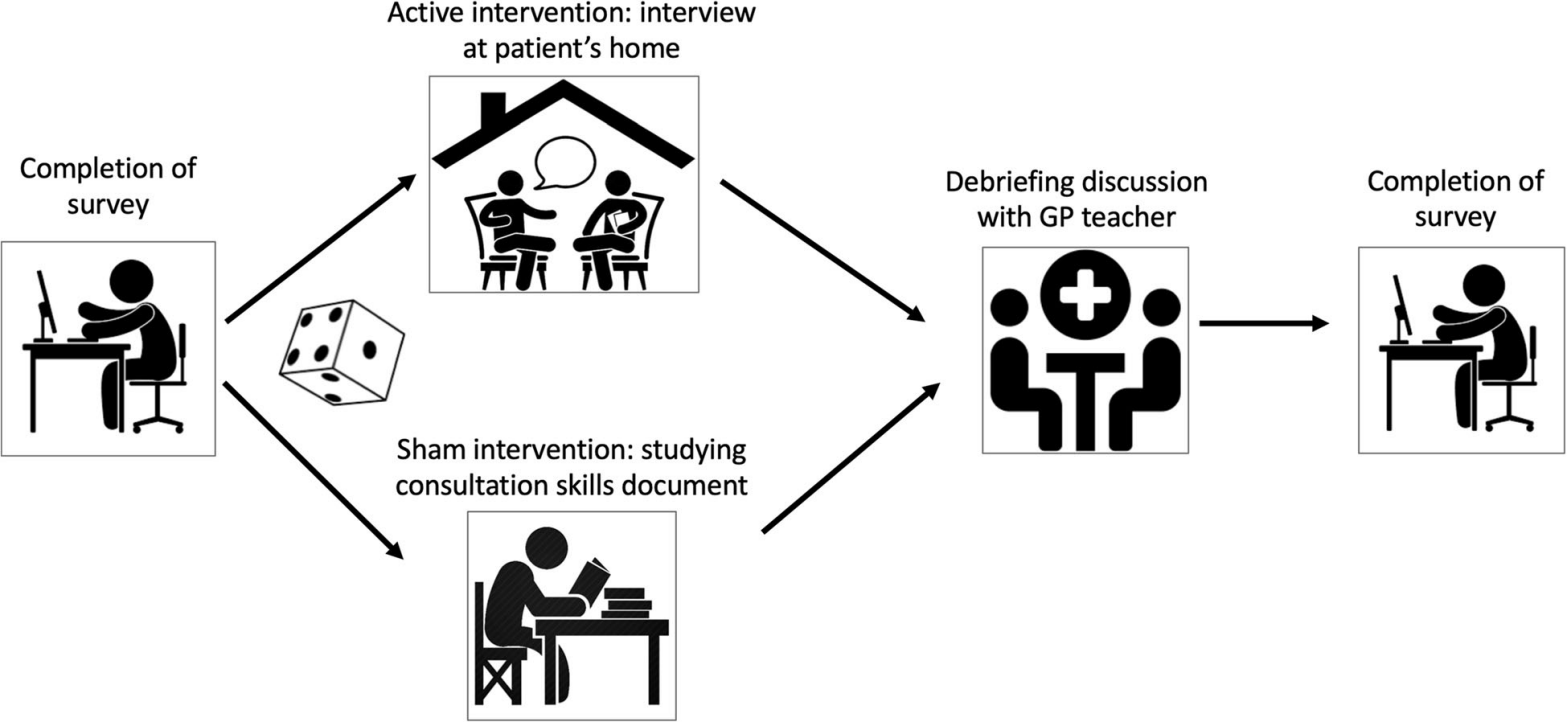
Diagram showing flow of participants through the study

The study is represented diagrammatically in Fig. 1.

### Development of the interventions

We designed the active intervention after reviewing and discussing the existing literature. Students were asked to visit a chronically ill patient in their own home and conduct an in-depth interview. They were given an interview guide, which helped students to elicit their patients’ views on their illnesses, and to ask about the physical, psychological and social effects that were a consequence of them. It gave open-ended questions that invited patients to talk about their lives and let students get to know their patients and their role in their families, as these have been found to be key in encouraging patient-centeredness [24]. Questions that encouraged a story-telling approach were used; this has been found to encourage patient-centred practice, by allowing clinicians to learn more about the patient as an individual, and to gain a better understanding of their personal meanings, experiences, and attitudes [25]. The sections of the interview guide were designed to map across to Mead and Bower’s conceptual framework of patient-centredness [26]. As the students were in their first year, so were mainly young and had little or no experience in interviewing patients, we designed the guide to make it easy for students to use it as a script. It was structured to ensure that they would cover the pertinent topics and use open-ended questions. The interviews were designed to last 60–90 min. The patient visits were followed by a 30-min student/GP teacher debriefing discussion, a process which helps learners make sense of their experience and reflect on their practice [27–29]. A proforma for the debriefing with the GP teacher prompted the students to summarise their consultation, reflect on their view of the patient as a person, and discuss what they learnt from the process, promoting reflective observation and abstract conceptualization, two core components of Kolb’s learning cycle [30].

Six medical students piloted the intervention with patients selected by their GP teachers and then participated in a focus group, led by RF, to discuss how well the interview guide worked and any organisational problems that they had encountered. The focus group was audio-taped and transcribed verbatim; thematic analysis was used to provide a descriptive thematic summary. Where the focus group identified weaknesses in the organisation of the patient interviews or in the patient interview guide, we used our findings to improve the guide and the information sheets for patients, students and GP teachers. Three medical students then piloted the updated interview guide, with one of the researchers (A-LC) role-playing the patient. The students gave feedback and recommendations on how the guide could be further improved. The pilots focussed on the practical aspects of implementing the intervention, including the ability of the intervention to promote patient-centeredness by letting students elicit their patients’ views on their illnesses and the effects on them, and getting patients to talk about their lives and the role they play in their families [23]. Taking these findings into account, RF, A-LC and MH further revised and finalised the patient interview guide (available from http://www.tinyurl.com/patient-centredness). The interview guide and instructions on how to organise the interview, including advice on students’ personal safety, were sent to students and their GP teachers before the students’ final visits to their teaching practices.

For the sham intervention, MH wrote a 1850-word (9 page) self-study guide called ‘Communication skills for medical students’ (available from http://www.tinyurl.com/patient-centredness). We sent it, with instructions on how to use it, to students and their GP teachers before the students’ final visits to their teaching practices.

### Sample size

Considering data from the literature [24], we powered the study to detect a mean between-group difference in PPOS-D12 scores of 0.16, with an SD of 0.42 and an effect size of 0.38. To detect this difference, with a power of 80% and a significance level of 5%, we calculated that 218 students would be needed, with a minimum of 109 in each group. To allow for a 20% drop-out rate, we therefore aimed to enrol a minimum of 275 students.

### Randomisation

We sent a list of all study participants to a central University agency (Clinical Trials Unit, University of Bern) where researchers used simple random sequences generated in the IBM SPSS (Version 22) statistical package to allocate participants, without stratification, to either the active or the sham intervention.

### Blinding

The researcher who performed the randomisation was blinded to the purpose of the interventions. To minimise performance and other reporting biases, we told students that they were randomised to one of two intervention groups, but we did not reveal that one was a sham comparator. We took the same approach with information sent to their GP teachers.

### Statistical analysis

We converted the PPOS-D12 survey Likert scale answers to a numerical score, ranging from 1 (‘I completely agree’) to 6 (‘I completely disagree’). For all the survey statements, ‘I completely agree’ was the most doctor-centred answer and ‘I completely disagree’ was the most patient-centred. For each respondent, the PPOS-D12 score was the mean of the scores for the twelve statements, so the lowest possible mean score (most doctor-centred) was 1, the highest possible mean score (most patient-centred) was 6.

For the primary outcome measure, to adjust for a difference in baseline PPOS-D12 scores between the two intervention groups, and after exploration of the data suggested that the effect of the baseline scores was linear, we compared the mean difference in the study start and end PPOS-D12 scores for the active and sham intervention groups using analysis of covariance (ANCOVA).

To measure secondary outcomes, we used linear regression to determine the effect of students’ baseline characteristics on PPOS-D12 scores at start of study, and a paired *t* test to determine the mean change over time of all student’ PPOS-D12 scores.

For the nested study, we used a paired *t* test to assess the difference between GP teachers’ PPOS-D12 scores and their students’ scores. We used the Pearson correlation coefficient to measure the association between GP teachers’ PPOS-D12 scores and changes in their students’ scores.

## Results

**Fig. 2:**
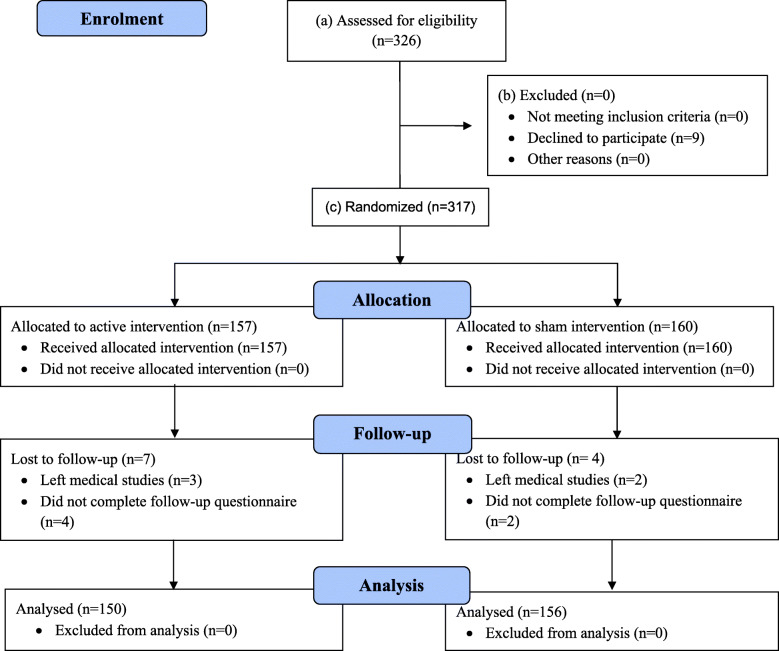
CONSORT 2010 Flow Diagram

Of the 326 students eligible for the study, 317 agreed to participate. On randomisation, 157 were allocated to the active intervention (patient interview), and 160 to the sham intervention (communication skills document), of which 150 (95.5%) and 156 (97.5%) students respectively completed both the study start and end PPOS-D12 surveys. All students received their allocated intervention. Eleven students were lost to follow-up (see CONSORT diagram, Fig. 2).

### Baseline characteristics

Of the 306 students who completed both surveys, 195 (63.7%) were women (Table 1). In the first survey, 102 (33.3%) indicated that they had experienced a serious, chronic illness in themselves, a friend or a close relative; 36 (11.8%) had studied another subject as an undergraduate before they started studying medicine; 108 (35.3%) had had contact with patients before they started at medical school. The mean PPOS-D12 score of all students combined was 4.19 at the start of the study. Despite the random allocation, we noted a significant difference between PPOS-D12 scores in the two groups at the start of the study (active intervention: 4.25, sham intervention: 4.14, *P* = 0.031).

**Table 1:** Students’ characteristics and mean PPOS-D12 scores at start of study

Characteristic	All participants completing both surveys (*n* = 306)	Allocated to active intervention (*n* = 150)	Allocated to sham intervention (*n* = 156)
Female, number (%)	195 (63.7)	92 (61.3)	103 (66.0)
Personal experience of a serious chronic illness, number (%)	102 (33.3)	55 (36.7)	47 (30.1)
Had previously studied another subject as an undergraduate, number (%)	36 (11.8)	19 (12.7)	17 (10.9)
Contact with patients before starting at medical school, number (%)	108 (35.3)	59 (39.3)	41 (31.4)
Baseline PPOS-D12 score^*^, mean (SD)	4.19 (0.47)	4.25 (0.44)	4.14 (0.50)

^*^ The lowest possible PPOS-D12 score (most doctor-centred) was 1, the highest possible score (most patient-centred) was 6

### Active intervention group: type of chronic disease and site of interview

Of the students allocated to the active intervention, 138 (92.0%) visited their patients at home or in residential accommodation; 11 (7.3%) saw their patients in their GPs’ practice; 76 (50.7%) interviewed patients with one of the four specified chronic diseases, and all but one of the other students saw patients with another significant chronic condition (Table 2). All students saw their GP teachers for a debriefing discussion.

**Table 2:** Site of interview and type of chronic disease

	Number (%)
Site of interview	
Patient’s home	125 (83.3)
Other residential accommodation	13 (8.7)
GP practice	11 (7.3)
Another site	1 (0.7)
Type of chronic disease	
Ischaemic heart disease	27 (18.0)
Chronic low back pain	21 (14.0)
Major depressive disorder	6 (4.0)
Chronic obstructive pulmonary disease	22 (14.7)
Chronic neurological disease	18 (12.0)
Chronic musculoskeletal disease	14 (9.3)
Diabetes	11 (7.3)
Congenital illness	7 (4.7)
Multimorbidity	5 (3.3)
Cancer	5 (3.3)
Cardiovascular disease	4 (2.7)
Other serious chronic illnesses	9 (6.0)
Not known	1 (0.7)

Of the students allocated to the sham intervention, one (0.6%) did not see their GP teacher for a debriefing discussion.

### Overall change in students’ PPOS-D12 scores

The mean PPOS-D12 score for both intervention groups combined was 4.19 (SD 0.47) at the start of the study, and 4.47 (SD 0.47) after the interventions, an increase of 0.27 (SD 0.44, *P* < 0.001), indicating a significant increase in patient-centred attitudes during the study.

### Comparison between the active and sham intervention groups

The PPOS-D12 scores rose by 0.23 for the active intervention group and by 0.32 for the sham intervention group (*P* < 0.001 for both groups). After adjusting the different baseline scores with ANCOVA, we found no significant difference in PPOS-D12 score changes between the two groups (*P* = 0.426) (Table 3).

**Table 3:** Change in students’ PPOS-D12 scores during the study

	All participants completing both surveys (*n* = 306)	Allocated to active intervention (*n* = 150)	Allocated to sham intervention (*n* = 156)	Significance level^*^
Increase in mean PPOS-D12 score (SD)	0.27 (0.44)	0.23 (0.41)	0.32 (0.47)	*p* = 0.426

* For difference between active and sham intervention groups, after adjustment for different baseline PPOS-D12 scores using ANCOVA

### Effect of baseline characteristics on PPOS-D12 scores at the start of the study

Our regression analysis revealed two characteristics that significantly predicted variance in the PPOS-D12 scores at start of study (Table 4). Being a woman (*P* = 0.001) and contact with patients before medical school (*P* = 0.032) were both associated with higher, more patient-centred, scores. Neither experience of a serious chronic illness in the students themselves, a friend or a close relative, nor history of previous undergraduate experience, were significant predictors of students’ PPOS-D12 scores at the start of the study (*P* = 0.646 and *P* = 0.158 respectively).

**Table 4:** Linear regression analysis of effect of student baseline characteristics on PPOS-D12 scores at start of study

Baseline characteristic	PPOS-D12 score (SD)	β-coefficient (95% CI)	*P* value
Gender			
Female	4.25 (0.43)	0.203 (0.086 to 0.315)	0.001^*^
Male	4.08 (0.51)		
Experience of a chronic illness in the students themselves or a close relative			
Yes	4.22 (0.48)	0.027 (−0.079 to 0.127)	0.646
No	4.18 (0.47)		
Had studied another subject as an undergraduate before starting to study medicine			
Yes	4.37 (0.54)	0.108 (−0.061 to 0.373)	0.158
No	4.17 (0.46)		
Contact with patients before going to medical school			
Yes	4.30 (0.48)	0.126 (0.011 to 0.238)	0.032^*^
No	4.13 (0.56)		

^*^ Significant at *p* < 0.05

### GP teachers’ PPOS-D12 scores

GP teachers’ PPOS-D12 scores (4.58) were significantly higher (more patient-centred) than their students both before the interventions, *P* < 0.001, and afterwards, *P* = 0.002 (Table 5). There was no correlation between the change in students’ PPOS-D12 scores and the scores of their GP teachers (*r* = 0.088, *P* = 0.138).

**Table 5:** Comparison of PPOS-D12 scores of students and their GP teachers

	Mean PPOS-D12 score (SD)
Students at start of study	4.19 (0.47)
Students at end of study	4.47 (0.47)
GP Teachers	4.58 (0.56)

## Discussion

### Principal findings

While first-year medical students became more patient-centred over the course of their longitudinal clerkship in primary care, the addition of a home-based interview with a chronically ill patient did not increase the effect.

### Interpretation of the results

An increase in first-year medical students’ patient-centred attitudes has not been described before, and this rise may be due to the longitudinal primary care clerkship that is embedded in University of Bern’s programme. Despite the careful development and successful implementation of the active intervention, the active intervention had no independent effect on students’ patient-centred attitudes.

Our finding that GP teachers had higher PPOS-D12 scores than their students may indicate a trend towards patient-centredness over the long-term, perhaps as a result of increasing experience, or it may be that doctors with patient-centred attitudes are more attracted to working in general practice. However, we found no association between individual GP teachers’ levels of patient-centredness and the degree of change in attitude of their allocated students.

### Comparison with existing literature

We did not expect an increase in students’ patient-centred attitudes, because other studies had found a decrease in patient-centred attitudes in students during their medical training. For example, in the USA, medical students’ PPOS scores decreased, falling from 4.61 in Year 1 to 4.46 in Year 4 [7]; Greek students’ PPOS scores reduced from 3.96 in Year 4 to 3.81 in Year 6 [8]; and South African students’ PPOS scores reduced from 2.65 in Year 1 to 2.25 in Year 6, with the most pronounced decrease in the first 2 years of study [9].

The University of Bern has a well-established, mandatory primary care clerkship that students begin in their first year, giving them immediate contact with patients, and this clerkship may increase the likelihood that students will develop more patient-centred attitudes. It is known that early patient exposure can have a positive effect on patient-centred attitudes [12], and longitudinal-integrated clerkships increase these attitudes regardless of specialty [31].

### Strengths and weaknesses of the study

The active intervention was developed iteratively, with two pilot studies and rounds of improvement to the patient interview guide. We had a very high response rate (96.8%) to our surveys with little loss to follow-up among students due to a rigorous recall scheme with several e-mail and phone reminders. All students who completed the study received their assigned intervention. Most in the intervention group (92.0%) complied with the instruction to conduct the interview at the patient’s residence. While only half of their students interviewed patients who had one of the four chronic conditions specified by the study team, all but one of the other students saw patients with other significant chronic conditions. Also, since patients and doctors may not always agree on which chronic condition is the primary diagnosis [32], it is possible that some of these patients did have one of the four specified conditions but did not see it as their primary problem or describe it that way to the students.

The significant imbalance between baseline PPOS-D12 scores in the two intervention groups was likely to be due to chance, as randomisation was performed independently and we detected no irregularities. We did not collect data on whether the students used the questions and topics given in the interview and debriefing guides. It may be that the single interview was too small an intervention to have a measurable effect, and that increasing the number of these would have produced a different outcome. While it is possible that the increase in PPOS-D12 scores was due to students’ desire to have a socially desirable score, this seems unlikely as they had no other clinical experience or teaching in in their first year other than that described in the paper, and were unlikely to have formed an opinion on the social desirability of certain responses.

### Implications for research and practice

We now know that medical students do not necessarily become less patient-centred over time, but we need to determine whether the increase in patient-centred attitudes in this study was produced by patient encounters during the primary care clerkships or by some other factor.

## Conclusions

Patient-centred attitudes increased during medical students’ first year of medical studies, possibly because of their longitudinal primary care clerkship. Being a woman, and contact with patients prior to medical school, were associated with higher levels of patient-centred attitudes in students at baseline. However, as a single, structured, home-based interview with a chronically ill patient had no additional effect, we do not recommend this intervention as a way to influence patient-centred attitudes. GP teachers tend to be more patient-centred than their allocated medical students,

## Supplementary information

**Additional file 1.**

## Data Availability

The datasets generated during and/or analysed during the current study are available from the corresponding author on reasonable request.

## Abbreviations

ANCOVA: Analysis of covariance
DALY: Disability-adjusted life years
GP: General Practitioner
PPOS: Patient-Practitioner Orientation Scale
SD: Standard Deviation
