# Supplementary material for: Does a home-based interview with a chronically ill patient help medical students become more patient-centred? A randomised controlled trial
Source: BMC Med Educ. 2020 Jul 11;20:217. doi: 10.1186/s12909-020-02136-y (PMC7353797; doi:10.1186/s12909-020-02136-y)
Supplement: Supplementary file 1 — Additional file 1. [file 12909_2020_2136_MOESM1_ESM.docx]

# Appendix. PPOS-D12 English translation

Possible Likert-scale responses: ‘I completely agree’; ‘I agree’; ‘I agree slightly’; ‘I disagree slightly’; ‘I disagree’; ‘I completely disagree’.

1. Although treating patients today is not so personal anymore, it is ultimately a small price for medical advancement.

2. The most important part of the normal visit is the physical examination.

3. Patients should rely on the knowledge of their doctors and not try to inform themselves about their condition.

4. When doctors ask a lot of questions about a patient's personal situation, they are too involved in private matters.

5. When doctors are really good at diagnosis and therapy, their treatment of patients is not so important.

6. Many patients keep asking questions without really learning anything new.

7. Patients tend to want to hear that everything is good, rather than real information about their health.

8. When doctors first try to be open and make a warm-hearted impression, they will not be so terribly successful.

9. If patients disagree with their doctors, it shows that they disrespect their doctors and do not trust them.

10. The patient must always be aware that the doctor is responsible.

11. It is not so important to know the cultural background of a patient and his life situation in order to treat his illness.

12. When patients get their own medical information, it often confuses them more than it helps them.
